# Frequent contiguous pattern mining over biological sequences of protein misfolded diseases

**DOI:** 10.1186/s12859-021-04341-y

**Published:** 2021-09-11

**Authors:** Mohammad Shahedul Islam, Md. Abul Kashem Mia, Mohammad Shamsur Rahman, Mohammad Shamsul Arefin, Pranab Kumar Dhar, Takeshi Koshiba

**Affiliations:** 1 Information Communication Technology Centre, Bangabandhu Sheikh Mujibur Rahman Maritime University, Pallabi, Mirpur-12, Dhaka, Bangladesh; 2 Department of CSE, Bangladesh University of Engineering and Technology, Dhaka, Bangladesh; 3 Faculty of Information Technology, Monash University, Clayton, VIC-3800 Australia; 4 Department of CSE, Chittagong University of Engineering and Technology, Raozan, Bangladesh; 5 Waseda University, 1-6-1 Nishiwaseda, Shinjuku-ku, Tokyo, 169-8050 Japan

**Keywords:** Amino acid, Association rule, Disease, Frequent pattern, Protein misfolding, Protein sequence

## Abstract

**Background:**

Proteins are integral part of all living beings, which are building blocks of many amino acids. To be functionally active, amino acids chain folds up in a complex way to give each protein a unique 3D shape, where a minor error may cause misfolded structure. Genetic disorder diseases i.e. *Alzheimer, Parkinson,* etc. arise due to misfolding in protein sequences. Thus, identifying patterns of amino acids is important for inferring protein associated genetic diseases. Recent studies in predicting amino acids patterns focused on only simple protein misfolded disease i.e. *Chromaffin Tumor*, by association rule mining. However, more complex diseases are yet to be attempted. Moreover, association rules obtained by these studies were not verified by usefulness measuring tools.

**Results:**

In this work, we analyzed protein sequences associated with complex protein misfolded diseases (i.e. *Sickle Cell Anemia, Breast Cancer, Cystic Fibrosis, Nephrogenic Diabetes Insipidus,* and *Retinitis Pigmentosa 4*) by association rule mining technique and objective interestingness measuring tools. Experimental results show the effectiveness of our method.

**Conclusion:**

Adopting quantitative experimental methods, this work can form more reliable, useful and strong association rules i. e. dominating patterns of amino acid of complex protein misfolded diseases. Thus, in addition to usual applications, the identified patterns can be more useful in discovering medicines for protein misfolded diseases and thereby may open up new opportunities in medical science to handle genetic disorder diseases.

## Introduction

Frequent Patterns (FP) are small patterns that repeatedly occur in a database, specially high in bio-sequences. The challenging task in pattern finding of bio-sequences is to find FP [1]. Data Mining has recently increased its popularity in classifying the biological sequences and structures based on their critical features and functions [2].

To survive, all living being need proteins, either in muscles or in cell membrane. Protein is one among the important factors and acts as constituents of all living organisms [2]. Protein is building blocks of hundreds of Amino acids joined together by peptide bonds. To be functionally active, amino acids chain folds up in complex way to give each protein a unique 3D shape. Protein folding is crucial for living organism as it affects gene skeleton. A small error in the folding process results in a misfolded structure, which can sometimes be lethal [3]. Protein misfolding is believed to be one of the primary causes of genetic disorder diseases such as Alzheimer’s disease, Parkinson’s disease, Huntington’s disease, Sickle cell anemia, Cystic fibrosis, Cancer and many other degenerative and neurodegenerative disorders [4]. Protein misfolding may occur due to an unwanted mutation in their amino acids or because of an error in the folding process. Thus, the relationship between these amino acids is very vital in case of protein misfolded diseases.

Frequent pattern mining is helpful to find the recurring relationships, association and correlation in a given data set [1]. Patterns can be represented as association rules and association rules are said to be strong if it satisfies both a minimum support threshold and a minimum confidence threshold. Therefore, frequent pattern mining can provide the solution for association rules formation among the most dominating amino acids for different protein misfolded diseases. To the best of our knowledge, three studies [2, 5, 6] have been identified on this issue. But all these were focused to predict pattern and association rules of the most dominating amino acids which cause the *Chromaffin Tumor* disease only. However, predicting the pattern and associations between more complex diseases are yet to be attempted in literature. Moreover, association rules obtained by these studies were not verified by usefulness measures.

The aim of this paper was to analyze protein sequences associated with complex protein misfolded diseases (i.e. *Sickle Cell Anemia*, *Breast Cancer, Cystic Fibrosis, Nephrogenic Diabetes Insipidus* and *Retinitis Pigmentosa-4*) and identify frequent patterns among their amino acids. Here, association rule mining was used to predict patterns. Association rules were considered to be strong if it had satisfied a minimum support and a confidence threshold. Then only useful rules were finally sorted out with the use of interestingness measures (i.e. *Lift*, *Bi-lift*, *Bi-improve* and *Bi-confidence*). Adopting quantitative experimental method, this work forms more reliable and strong association rules among the most dominating amino acids of corresponding proteins and identify the dominating patterns of amino acid of complex protein misfolded diseases. Identification/reporting of such variant of amino acids for those particular five genetic diseases may have versatile implications. An improved capacity in identifying the relations among the most dominating amino acids in protein sequences related to disease will have an immediate impact on the diagnosis, treatment, and prevention of genetic disorders and thus may open up new opportunities in medical science to handle the concerned genetic disorder diseases.

This paper is organized as follows. “Theoretical framework” section presents theoretical background of related issues. “Literature review” section highlights an overview of the related works. The experimental design is presented in “Methodology” section and “Experimental results” sectionrepresents the data analysis and results. In “Comparison with previous studies” section some comparative analysis with previous studies has been made. Potential implications of the finding of this work are focused in “Implication of the findings” section. The concluding remarks and the future work are presented in the final section.

## Theoretical framework

Some of the concepts and issues such as protein structure, protein associated diseases, association rule mining and their interestingness measures which have been considered in this paper are discussed below.

### Amino acid and protein

**Fig. 1:**
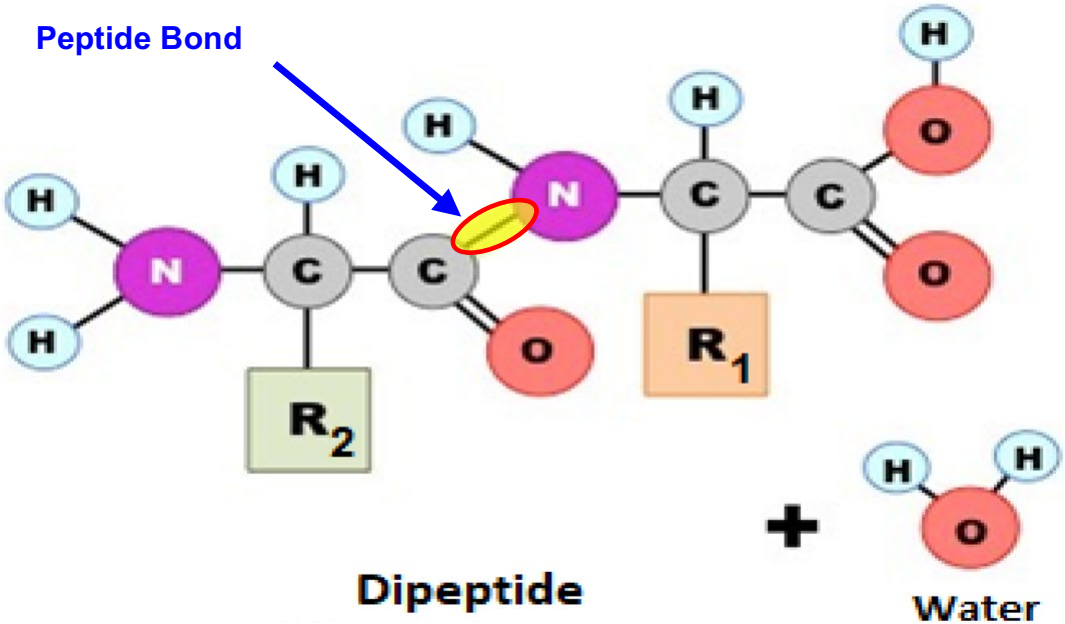
Amino Acids joined together through *Peptide Bonds*

To survive, all living being needs proteins. The biological activity of the protein is determined by the chemical properties of the amino acids. Amino acids are made from carbon, hydrogen, nitrogen and oxygen. Though more than 50 amino acids have been discovered; only 20 are used to make proteins in human body. These 20 amino acids convey a vast array of chemical versatility within proteins [7]. Proteins are complex molecules, made up of hundreds of amino acids that are attached to one another by peptide bonds (Fig. 1), forming a long chain [8]. Amino acids sequences contain the necessary information, basing on which, protein determine how that protein will fold into a 3D structure and the stability of the resulting structure.

### Protein misfolding

**Fig. 2:**
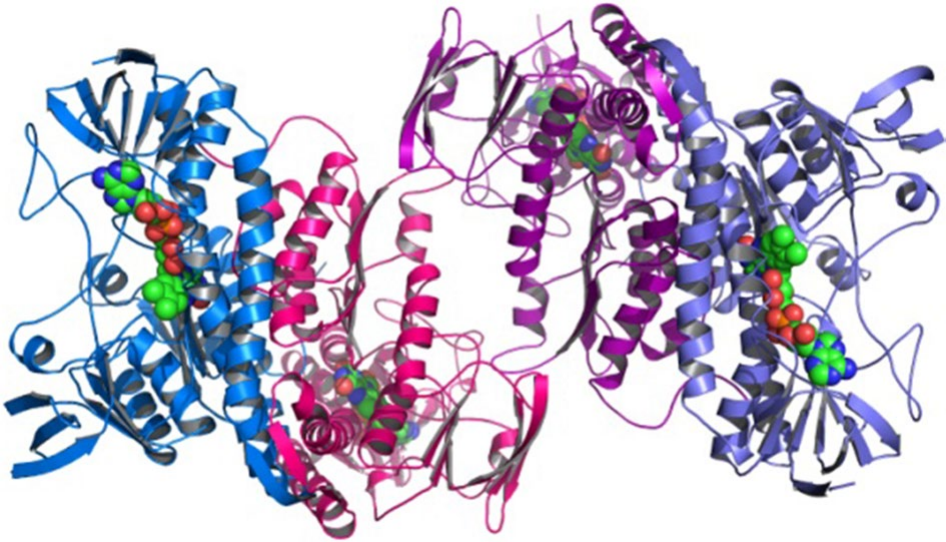
Quaternary/final protein structure

A protein can be functionally active when it acquires a unique 3D conformation through the complicated folding of the polypeptide chain coded from the nuclear genome (Fig. 2). Protein may have adverse effect on its functionality if not folded properly. Proteins that are not able to achieve native state, due either to an unwanted mutation in their amino acid sequence or simply because of an error in folding process, are recognized as misfolded.

### Protein misfolding diseases

For the last couple of years, protein misfolding and its effects have become a matter of great concern. According to the prion researcher Susan Lindquist, ‘protein misfolding could be involved in up to half of all human diseases’ [9]. Many cancers and other protein-misfolding disorders are caused by mutations in proteins. Protein misfolding is believed to be the primary cause of genetic disorder diseases such as *Alzheimer*, *Parkinson*, *Huntington*, *Sickle cell anemia*, *Cystic fibrosis*, *Cancer* and many other degenerative and neurodegenerative disorders [4]. Over last two decades, protein misfolding and its pathogenic effect have become a significant area of human bio-molecular research. In this work, five protein misfolded diseases (i.e. *Sickle Cell Anemia* [10], *Breast Cancer* [11]*, Cystic Fibrosis* [12]*, Nephrogenic Diabetes Insipidus* [13] and *Retinitis Pigmentosa 4* [14]) have been experimented.

### Frequent pattern mining in bioinformatics

Frequent patterns are either itemsets or subsequences or substructures which appear in a data set with a frequency that is equal to or higher than a threshold specified by the user. Data mining can be the most active technique to infer structure and principles of biological datasets and to solve biological problems. Pattern mining is useful in bioinformatics for predicting rules of certain elements in genes, for protein function prediction, for gene expression analysis, for protein fold recognition and for motif discovery in DNA sequences [13]. Thus, frequent pattern mining can be used to find recurring relationships, association and correlation between amino acids for protein misfolded diseases.

### Association rule mining

Association rule mining is one sorts of pattern mining which is built from frequent itemset mining. In data mining, association rule learning is a popular and well researched method for discovering interesting relations between variables in large databases [15]. Patterns can be represented as association rules and the association rules are said to be strong if it satisfies both a minimum support threshold and a minimum confidence threshold. Therefore, frequent pattern mining can provide solution for association rules formation among the most dominating amino acids for different protein misfolded diseases. To analyse, predict and manage bulk biological data, numerous computer algorithms and methods are developed which help to compare and align biological sequences and predict bio-sequence patterns [1]. In this work, as tools of association rule mining, Apriori algorithm was used to analyse, predict and identify desired pattern of dominating amino acids in the protein sequences.

### Interestingness measures for association rules mining

Association rules mining algorithm can generate a lot of association rules or patters or knowledge, but most of them have redundant information and limited resources. Therefore, it is essential to evaluate the interestingness (or usefulness) of the association rules before their practical use. In this work objective measures were used for evaluating the interestingness of the rules. Benefit of using objective measures is that they mainly use statistical methods and a quantitative value to determine the interestingness of rules which is reliable, easy to operate and convincing. Objective measures are *Support*, *Confidence*, *Lift*, *Improve*, *Validity*, *Influence*, *Conviction* and *Bi-lift*, *Bi-improve* and *Bi-confidence* for *Lift*, *Improve* and *Confidence*, respectively etc. [16].

Objective measures *support*, *confidence*, *lift* and *improve* [17] were used by Islam et al. [18] to generate and detect strong and interesting association rules.

1. ***Support***: The *support* of an itemset *X*, *supp (X)* is defined as proportion of transaction in data set in which the item *X* appears. It indicates popularity of an itemset.
  1 $$supp\left( X \right) = \frac{No. \,of\,transactions\,in\,which\,itemset\,X\,appeared}{{Total\,no.\,of\,transactions}}$$
2. ***Confidence***: The *confidence* of a rule is defined as:
  2 $$conf\left( {X \to Y} \right) = \frac{{supp\left( {X \cup Y} \right)}}{supp\left( X \right)}$$
3. ***Lift****:* The *lift* of a rule is defined as:
  3 $$lift\left( {X \to Y} \right) = \frac{{supp\left( {X \cup Y} \right)}}{supp\left( Y \right) * supp\left( X \right)}$$

The rule (*X* → *Y*) will be considered as positively correlated rule if its *Lift* value is greater than 1. Thus, those rules are useful only whose *Lift* value is greater than 1.

4. ***Improve****: Improve* is a relatively new interestingness measure method of association rules based on the description of the defects of the traditional interestingness measurement method and defined as:
  4 $$Improve \left( {X \to Y} \right) = \left[ {P(Y | X} \right) - P\left( Y \right)]$$

However, *Support*, *Confidence, Lift* and *Improve* have their own limitation.

1. ***Limitation of support and confidence*** Due to subjectively selected support threshold value, many infrequent itemsets which have been discarded may have potential value. The rules are called strong association rules if the *Support* and *Confidence* are larger than the respective minimum *support* and minimum *confidence* threshold. But strong association rules are not always effective, some are not what users are interested in, and some are even misleading [19].
2. ***Limitation of lift*** Lift takes events A and B in equivalence position. According to the *Lift*, (A → B) and (B → A) are the same; that means, if we accept rule (A → B), (B → A) should also be accepted, but fact is not like this [19].
3. ***Limitation of improve*** [20] Firstly, how much improvement of probability can be called improvement? Secondly, the probability of former pieces’ occurrence will seriously affect *Improve* evaluation in such a way that when it is high, the *improve* value will be very small all the time.

To overcome the shortcomings of *Lift*, *Improve* and *Confidence*, literature [19] suggests following corrections to the measures:

1. ***Bi-lift*** [19] The correction of *Bi-lift* measure method, $lift\left( {\overline{A} \to B} \right)$ as denominator, and *lif*(*A* → *B*) as numerator, namely, ratio of *lift*(*A* → *B*) to *lift*(*A* → *B*); *Bi-lift* formula is as follows:
  5 $$\begin{aligned} Bi - lift\left( {A \to B} \right) & = \frac{{lift\left( {A \to B} \right)}}{{lift\left( {\overline{A} \to B} \right)}} \\ & = \frac{{P\left( {AB} \right)/P\left( A \right)P\left( B \right)}}{{P\left( {\overline{A}B} \right)/P\left( {\overline{A}} \right)P\left( B \right)}} \\ & = \frac{{P\left( {AB} \right)/P\left( {\overline{A}} \right)}}{{P\left( {\overline{A}B} \right)/P\left( A \right)}} \\ \end{aligned}$$
  Its value range is [0, ∞]. The higher the *Bi-lift* (*A* → *B*), the better the rule *A* → *B* is.
2. ***Bi-improve*** Because of the defects of *improve*, the paper [19] put forward *Bi-improve*. In order to eliminate the influence, correction was given by multiplying the ratio of the occurrence possibility of antecedent to the no occurrence probability of antecedent. *Bi-improve* formula is as follows:
  6 $$\begin{aligned} Bi - improve\left( {A \to B} \right) & = \left[ {P\left( {B|A} \right) - P\left( B \right)} \right]* \frac{P\left( A \right)}{{P\left( {\overline{A}} \right)}} \\ & = \frac{{P\left( {AB} \right) - P\left( A \right) P\left( B \right)}}{{P\left( {\overline{A}} \right)}} \\ \end{aligned}$$
  The higher the *Bi*-*impro*v*e* (*A* → *B*), the better the rule *A* → *B* is.
3. ***Bi-confidence*** [19] The confidence of association rules only thinks about the occurrence possibility of “B” when “A” occurs, but not consider the relationship between “A” and “B” when “A” does not occur. So, it makes a lot of association rules mining invalid. For the above problems, concept of *Bi-confidence* is defined as follows:
  7 $$\begin{aligned} Bi - confidence \left( {A \to B} \right) & = \frac{{P\left( {AB} \right)}}{P\left( A \right)} - \frac{{P\left( {\overline{A}B} \right)}}{{P\left( {\overline{A}} \right)}} \\ & = \frac{{P\left( {AB} \right) - P\left( A \right)P\left( B \right)}}{{P\left( A \right) * \left[ {1 - P\left( A \right)} \right]}} \\ \end{aligned}$$
  The value range of *Bi-confidence* is [− 1, 1]. If the value of *Bi-confidence* is greater than 0, then *A* and *B* have positive correlation. If the *Bi-confidence* is equal to 1, then it shows that “A” and “B” in record set appear together or not. If the *Bi-confidence* is equal to 0, then “A” has no relation with “B”. If the *Bi-confidence* is less than 0, then it shows that “A” and “B” have the negative correlation. The higher the *Bi*-*confidence* (*A* → *B*), the better the rule *A* → *B* is.

## Literature review

Frequent Contiguous Patterns (FCP) are small patterns that repeatedly occurs in a database, specially high in bio-sequences. Biological sequences such as DNA and protein sequences consist of long linear chain of chemical components and typically contain a large number of items [21]. Frequent pattern mining is helpful to find the recurring relationships, association and correlation in a given data set [1]. In data mining, association rule learning is a popular and well researched method for discovering interesting relations between variables in large databases [15]. The challenging task in pattern finding of biological sequences is to find frequent contiguous patterns [1]. Data Mining has increased popularity in classifying biological sequences and structures based on their critical features and functions [2].

Protein is one among the important factors and acts as the constituents of all living organisms [2]. Protein misfolding is believed to be the primary cause of genetic disorder diseases such as Alzheimer’s disease, Parkinson’s disease, Huntington’s disease, Sickle cell anemia, Cystic fibrosis, Cancer and many other degenerative and neurodegenerative disorders [4]. Proteins are made up of smaller building blocks called amino acids, joined together in chains [22]. The chains of amino acids fold up in complex ways, giving each protein a unique 3D shape. Thus, relationship between these amino acids is very vital in case of protein misfolded diseases. Frequent pattern mining can provide the solution for association rules formation among the most dominating amino acids for different protein misfolded diseases. To the best of our knowledge, three studies [2, 5, 6] have been identified on this issue.

Lakshmi and Hariharan [5] aimed to predict patterns applying strong association rules over the frequent itemsets of the protein sequence named *Succinate dehydrogenase* which is involved in *chromaffin tumor* disease. The system generated frequent itemsets from the protein sequence and constructs a frequent pattern tree. Thereafter strong association rules were generated based on 90% confidence threshold to identify the dominating amino acids.

Lakshmi and Hariharan [2] conducted another similar research in finding the most dominating amino acids (in *Succinate dehydrogenase* protein) which causes the disease *chromaffin tumor.* Here, Apriori algorithm was used in finding frequent items using candidate generation and then generating association rules from those frequent itemsets. In predicting the pattern, this work considered 5 as minimum *Support* count and 90% *Confidence* threshold.

Dhumale carried out similar work [6] to find dominating amino acids responsible to cause five diseases, i.e. *Epilepsy*, *Hartnup*, *Cystinuria*, *Alzheimer* and C*hromaffin Tumor*. As deduction, the author claimed five amino acid patterns (association rules), each to be responsible for an individual diseases. This work suffers serious limitations. Firstly, the experimented protein sequence is anonymous. Secondly, all the mentioned diseases might not be associated with a single protein. The author did not provide any credibility of the information. Moreover, no authentic literature was found in this regard. It is to mention that all diseases are not associated with the protein changes. Some are multi-factorial diseases; some are infectious diseases and so on. Thirdly, the author arbitrarily increased the minimum *Support* count from 2 to 5, generated association rules with confidence threshold 90% and declared set of amino acid pattern (association rule) as responsible for each of the disease. But on what basis this deduction was arrived was not at all cleared.

The above three works were focused to predict the pattern and association rules of amino acids which causes the *Chromaffin Tumor* disease only. However, finding patterns of other protein associated diseases or more complex protein misfolded diseases ate yet to be attempted in the literature. Moreover, it is also important to predict interesting association rules for practical use. But association rules obtained by these studies were not verified by usefulness measures.

## Methodology

In this study, five protein misfolded diseases were taken in consideration. The protein sequences associated with each of the diseases were collected from a well-recognised protein data bank. Then the associative patterns among the amino acids were identified using a data mining technique. To generate the strong association rules from the amino acids of the protein associated diseases, support count were raged between 3 to 5 and minimum confidence as 90%. Based on the strong association rules, this proposed system was focused on predicting the most dominating amino acids than the other amino acids that cause the disease from the protein data sets.

### General work flow

**Fig. 3:**
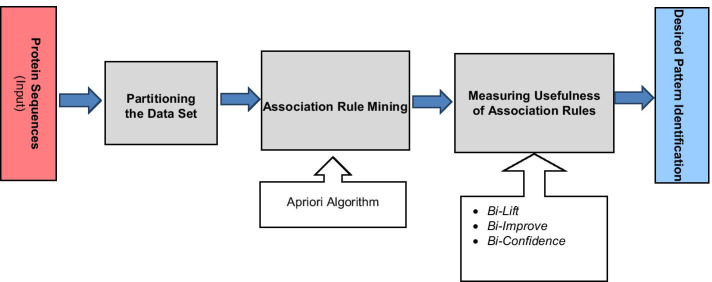
Architecture of the system

The proposed system works in five steps. General work flow of the proposed system is shown in Fig. 3.

*(1)***Selection of protein sequence** As stated earlier, in this work, five misfolded diseases (i.e. *Sickle Cell Anemia, Breast Cancer, Cystic Fibrosis, Nephrogenic Diabetes Insipidus* and *Retinitis Pigmentosa 4*) were taken in consideration. Protein sequences (amino acid chain) associated with these diseases were collected from protein data bank named Universal Protein Resource (www.uniprot.org/) in FASTA form. It is to note that the UniProt is a comprehensive resource for protein sequence and annotation data. The mission of UniProt is to provide the scientific community with a comprehensive, high-quality and freely accessible resource of protein sequence and functional information. Due to its world-wide acceptance and high degree of reliability, protein sequences for this work were collected from UniProt protein knowledgebase. Table 1 shows the experimented human diseases, their associated proteins and their lengths.

**Table 1:** Different human diseases and involved proteins

Disease	Protein name	Lengths
Sickle cell anemia	Hemoglobin Subunit Beta	147
	Entry Code: P68871	
Breast cancer	Breast Cancer Type 1 Susceptibility Protein	1863
	Entry Code: P38398	
Cystic fibrosis	Cystic Fibrosis Transmembrane Conductance Regulator (CFTR)	1480
	Entry Code: P13569	
Nephrogenic diabetes insipidus (NDI)	Vasopressin V2 Receptor (V2R)	371
	Entry Code: P30518	
Retinitis Pigmentosa 4 (RP4)	Rhodopsin (Opsin-2)	348
	Entry Code: P08100	

*Source:*http://www.uniprot.org/

*(2)***Partitioning data set** Each of the protein sequences (amino acid chain) were subdivided into amino acid sub sequences of length 10. For example, Hemoglobin Subunit Beta protein sequence (associated with *Sickle Cell Anemia* disease) contained amino acids of 147 length which was partitioned into 15 sub sequences of length 10 each as shown in Table 2.

**Table 2:** Sub sequences of hemoglobin subunit beta protein sequence

10	20	30
MVHLTPEEKS	AVTALWGKVN	VDEVGGEALG
40	50	60
RLLVVYPWTQ	RFFESFGDLS	TPDAVMGNPK
70	80	90
VKAHGKKVLG	AFSDGLAHLD	NLKGTFATLS
100	110	120
ELHCDKLHVD	PENFRLLGNV	LVCVLAHHFG
130	140	147
KEFTPPVQAA	YQKVVAGVAN	ALAHKYH

*Source*: http://www.uniprot.org/uniprot/P68871

*(3)***Association rule mining** The sub sequences of amino acids were then used for associative pattern identification through Apriori Algorithm data mining technique. Association rules were generated based on minimum support count threshold and minimum 90% confidence level. It is to mention that the value of the minimum support count is usually subjectively decided by the researchers. Higher the minimum support count, smaller and stronger the association rules for a particular confidence level. However, if the support count is too high then many interesting association rules may be discarded. In this work, the lengths of protein sequences were not uniform and thus to generate and analyse a significant number of association rules, the minimum support count was subjectively selected 3 for Hemoglobin Subunit Beta protein, 5 for Breast Cancer Type 1 susceptibility and Cystic Fibrosis Transmembrane Conductance Regulator proteins and 4 for Vasopressin V2 Receptor and Rhodopsin proteins.

*(4)***Measuring interestingness of association rules** In the previous steps, association rule algorithm would generate a significant number of rules. However, all these association rules may not be practically useful. Therefore, the interestingness of these rules were measured and evaluated. This evaluation would be conducted by objective or subjective measures. Considering the effectiveness and stability in results, improved objective measuring tools (i.e. *Bi-lift, Bi-improve* and *Bi-confidence*) were used to evaluate the association rules comprehensively. As such, *Bi-lift, Bi-improve* and *Bi-confidence* value of each of the association rules were calculated to finally prune the useful association rules.

*(5)***Identification of patterns** Based on the strong and useful association rules, this proposed system focused on predicting the most dominating amino acids, and thus the associative patterns among the amino acids were identified for each protein misfolded disease.

Combining *Support* and *Confidence* with *Lift*, *Bi-lift*, *Bi-improve* and *Bi-confidence*, a reasonable framework for identifying strong and interesting association rules was developed. In this work, the associative patterns among the amino acids were generated and measured by using following sequences:

1. Firstly, *Support* and *Confidence* threshold was used to filter out frequent itemsets and strong association rules
2. Secondly, *Lift, Bi-lift*, *Bi-improve*, and *Bi-confidence* value were calculated
3. Then, according to the *Bi-lift*, *Bi-improve* and the *Bi-confidence* value, useful association rules were sorted out

Actually, the final evaluation results of these three kinds of measure methods are very close and give perfect results.

### Algorithm

In this work, the algorithm used takes four inputs: (i) the protein sequence of a particular protein misfolded disease, (ii) minimum support count (iii) the threshold confidence level and (iv) usefulness measuring parameter. Then the algorithm returns the strong and useful association rules of the most dominating amino acids for the concerned protein misfolded disease. Pseudocode as follows:
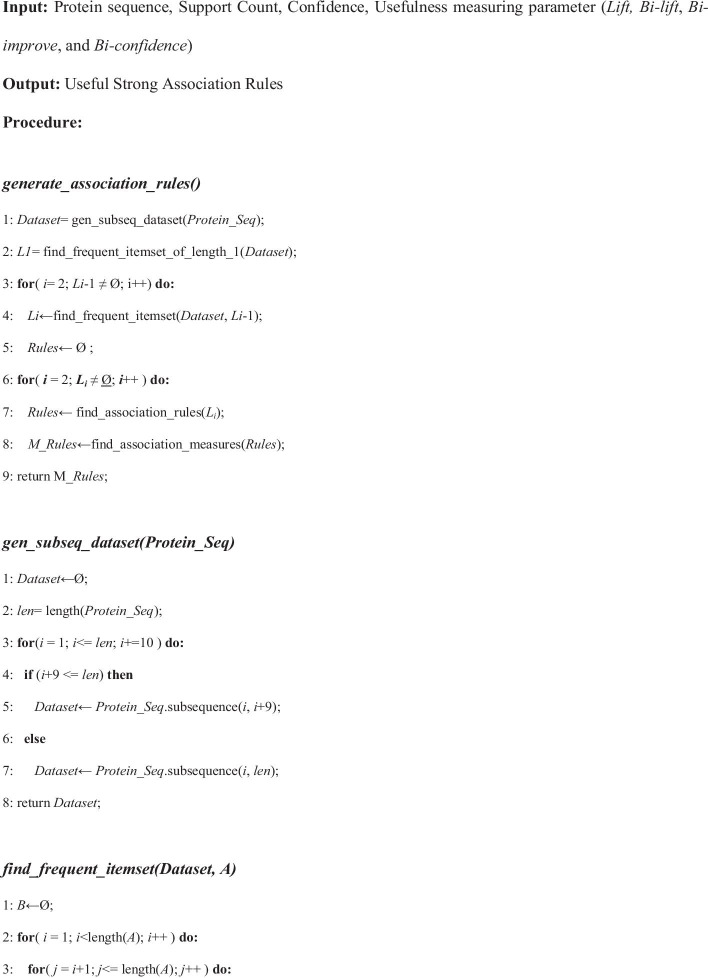

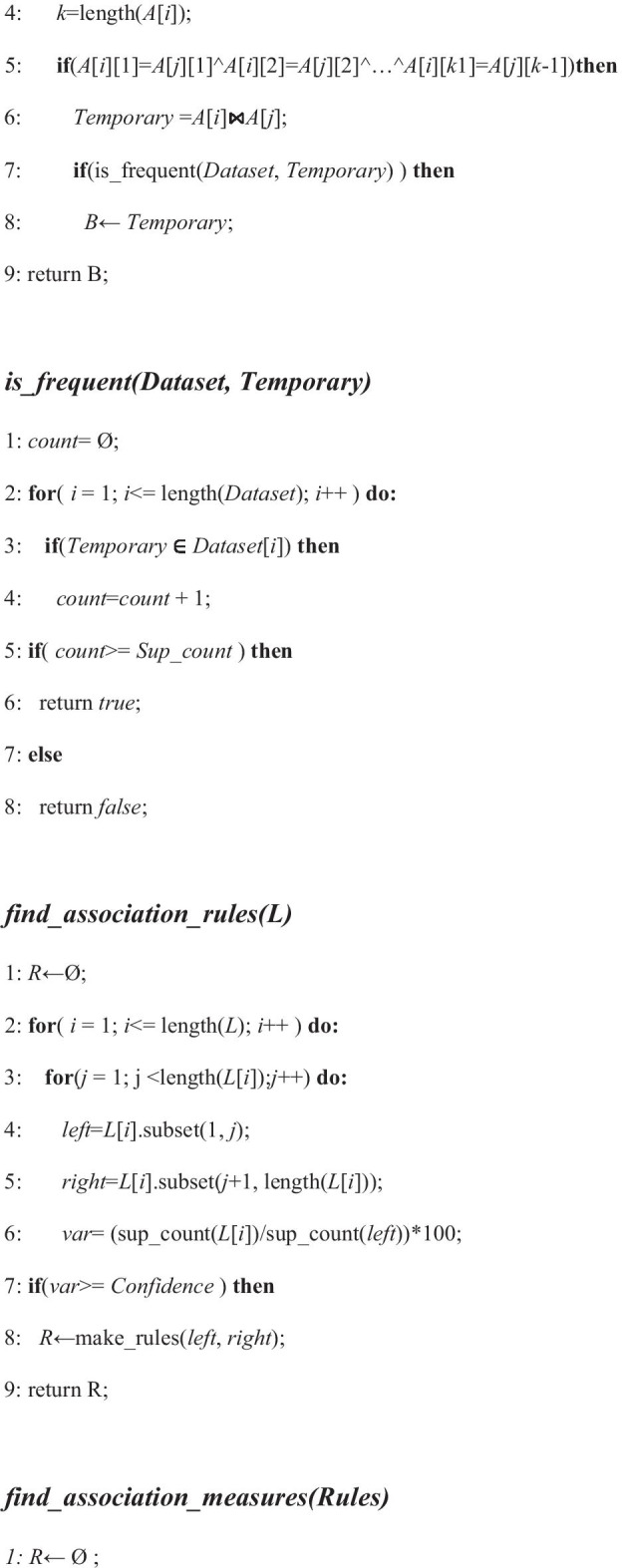

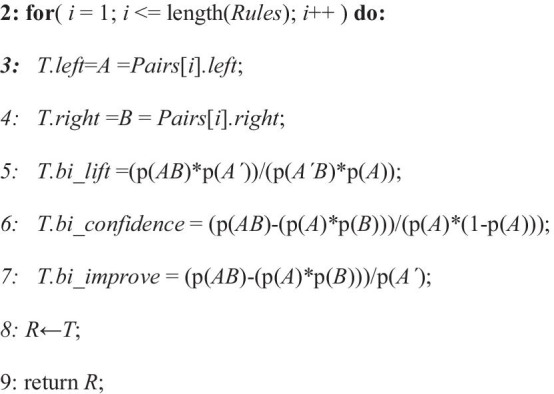


The procedure starts with the method *generate_association_rules().*

- ***Step-1*** In this step, the ***Dataset*** is generated by calling *gen_subseq_dataset*(***Protein_Seq***). This method splits the protein sequence after each 10 elements of the given misfolded protein sequence and insert them into the ***Dataset*** and return it.
- ***Step-2*** In this step, ***L***_***1***_ is generated which denotes the frequent itemset of length 1 by calling the method named *find_frequent_itemset_of_length_1* (***Dataset***).
- ***Steps-3, 4*** In this step, a loop runs until ***L***_***i***−1_ becomes empty. Here, ***L***_***i***_ denotes the ***i***th frequent itemset. ***L***_***i***_ is generated by calling *find_frequent_itemset*(***Dataset***, ***L***_***i***−1_). This procedure generates the ith frequent itemset from the (***i*** − 1)th frequent itemset. It runs a nested loop where it takes each two item from (***i*** − 1)th frequent itemset and if it matches all the protein except the last one between that two itemset, then it joins that two itemset and check if the itemset is frequent or not. If the itemset is frequent, then it insert that itemset into the ***i***th frequent itemset. After completing this procedure, it returns the ***i***th frequent itemset.
- ***Steps-6, 7*** In this step, a loop runs until ***L***_***i***−1_ becomes empty starting from ***L***_2_ and find the association rules by calling *find_association_rules*(***L***). In each iteration of the loop it takes an item from the ***i***th frequent itemset and splits it into two parts from first to last. Then it calculates the confidence and inserts the rules having confidence above the given confidence and returns the set of rules. Finally, the association rules are stored in ***Rules***.
- **Step-8** In this step, a loop runs over all items of ***Rules*** by calling *find_association_measures* (***Rules***). Then it calculates ***bi_lift***, ***bi_confidence*** and ***bi_improve*** for each of the items of ***Rules***. Finally, the rules with metrics for association rules measuring are stored in ***R***.

## Experimental results

The algorithm of the experiment had been implemented using C +  + in a laptop computer with an Intel Core i5-7200U CPU (clock frequency 2.7 GHz and 4 GB RAM). Experimental results were obtained from each of the protein sequences. During the computation, the number of iterations was not fixed. The algorithm was continued till no further successful extensions were found. The work thus followed three basic actions:

- Frequent itemsets generation
- Generation of strong association rules
- Identifying interesting/useful association rules

In doing so, following considerations were made:

- Support count threshold 3, 4 and 5 for frequent itemset generation.
- Minimum 90% confidence level to obtain strong association rules.
- Using *Lift, Bi-lift*, *Bi-improve* and *Bi-confidence* as measuring instrument to find useful strong association rules.

### Frequent itemsets generation

Frequent itemsets generation means the frequent amino acid sets generation from the transactional protein datasets (sub sequences). For every protein sequences, frequent itemsets were generated. The algorithm maintains list of frequent amino acid sets to further generate strong association rules.

**Fig. 4:**
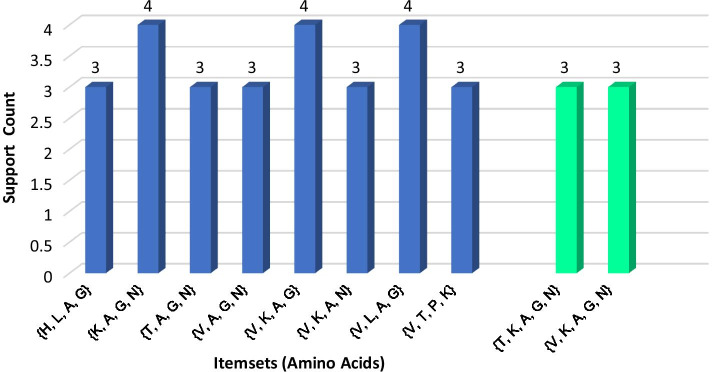
A few frequent 4-itemsets and 5-itemsets obtained from protein sequence for *Sickle Cell Anemia*

***(1) Disease-1: sickle cell anemia*** For *Sickle Cell Anemia*, protein sequence *Hemoglobin Subunit Beta* was loaded as input file. Here, 3 was considered as minimum support count. The process continued up to 5th iteration and garnered total 135 itemsets (comprising 1-itemsets to 5-itemsets) of amino acids. A few of the generated frequent itemsets for *Sickle Cell Anemia* is graphically represented in Fig. 4.

**Fig. 5:**
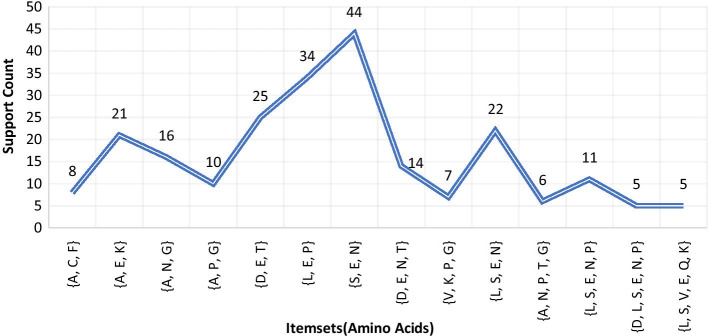
A few frequent 3 to 6-itemsets obtained from protein sequence for *Breast Cancer*

***(2) (Disease-2: Breast cancer*** For *Breast Cancer* disease, protein chain sequence *Breast Cancer Type 1 Susceptibility Protein* was loaded in the process as the input file. This protein chain sequence was consisted of total 1863 amino acids. Here, due to the long length, 5 was considered as the minimum support count. The process satisfied the threshold support count unto 6th iteration and generated total 1806 itemsets (comprising 1-itemsets to 6-itemsets) of amino acids. Among this, frequent 1-itemsets were 20 in number, frequent 2-itemsets were 176, frequent 3-itemsets were 669, frequent 4-itemsets were 744, frequent 5-itemsets were 191 and frequent 6-itemsets were 6. A concise list of frequent itemsets generated for this disease is shown in Fig. 5.

**Fig. 6:**
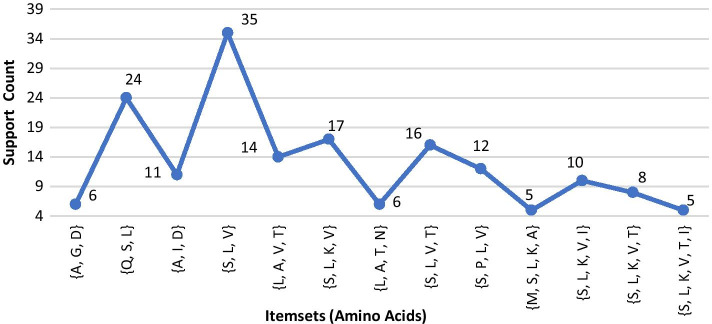
A few frequent 3 to 6-itemsets obtained from protein sequence for *Cystic Fibrosis*

***(3) Disease-3: Cystic fibrosis*** For *Cystic Fibrosis* disease, protein chain sequence *Cystic Fibrosis Transmembrane Conductance Regulator (CFTR)* (length 1480 amino acids) was loaded in the process as the input file. Here, due to long length, minimum support count 5 was considered. The process continued up to 6th iteration and garnered total 1464 itemsets (comprising 1-itemsets to 6-itemsets) of amino acids. Among this, frequent 1-itemsets were 20 in number, frequent 2-itemsets were 178, frequent 3-itemsets were 607, frequent 4-itemsets were 563, frequent 5-itemsets were 95 and frequent 6-itemsets were only 1. A concise list of frequent itemsets generated for this disease is shown in Fig. 6.

**Fig. 7:**
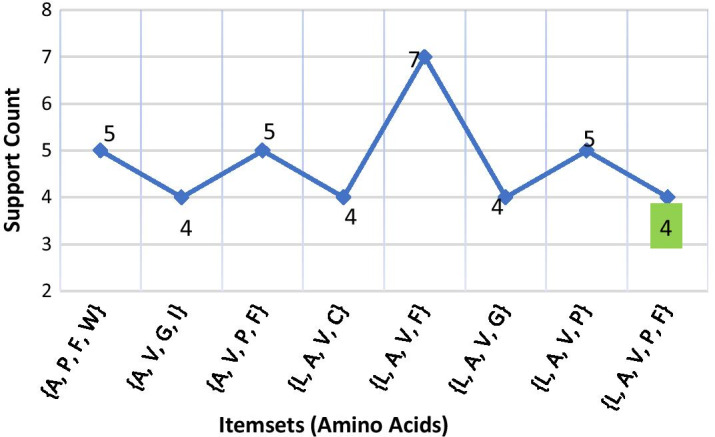
A few frequent 4-itemsets and 5-itemsets obtained from protein sequence for *Nephrogenic Diabetes Insipidus*

***(4) Disease-4: Nephrogenic diabetes insipidus*** For *Nephrogenic Diabetes Insipidus (NDI)*disease, protein sequence *Vasopressin V2 Receptor* was loaded as the input file. Here, due to moderate length (371), minimum support count 4 was considered. The process continued up to 5th iteration and generated total 234 itemsets. A few of generated frequent itemsets for *Nephrogenic Diabetes Insipidus* is shown in Fig. 7.

**Fig. 8:**
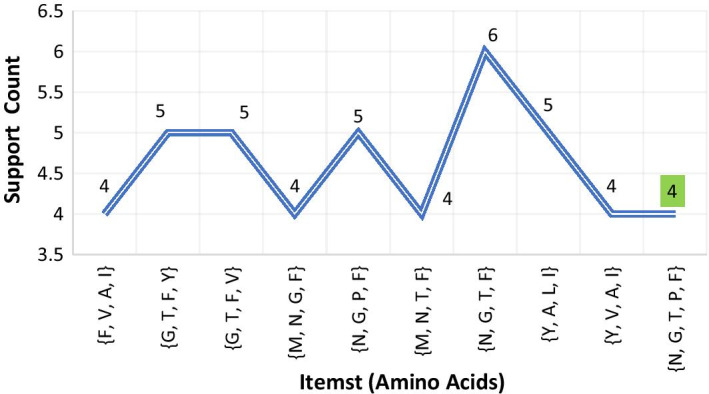
A few frequent 4-itemsets and 5-itemsets obtained from protein sequence for *Retinitis Pigmentosa 4*

***(5) Disease-5: Retinitis pigmentosa 4*** Protein sequence *Rhodopsin* *(Opsin-2)* was loaded in the process as input for *Retinitis Pigmentosa 4 (RP4)* disease. Here, 4 was considered as the minimum support count. The process continued up to 5th iteration and generated total 268 itemsets. Few generated frequent itemsets for *Retinitis Pigmentosa 4* is graphically represented in Fig. 8.

### Strong association rules generation

The algorithm maintains list of frequent itemsets (amino acid sets) for each protein sequence and from this list corresponding strong association rules are generated considering 90% confidence threshold in each case.

***(1) Disease-1: Sickle cell anemia****:* The process generated 698 association rules from 135 frequent itemsets. Among these rules, only 95 rules satisfied the minimum confidence level (90%) and were considered as accepted strong association rules and rest 603 rules were rejected. Examples of few association rules in this phase are shown in Table 3.

**Table 3:** Generation of association rules for *sickle cell anemia*

Ser	Assoc rule	Conf	Result	Ser	Assoc rule	Conf	Result
1	A → D	20%	Rejected	492	G → AKT	23%	Rejected
2	D → A	43%	Rejected	493	GK → AT	60%	Rejected
·	·	·	·	494	GKT → A	100%	Accepted
·	·	·	·	495	GT → AK	100%	Accepted
146	G → AK	39%	Rejected	·	·	·	·
147	GK → A	100%	Accepted	694	KNV → AG	100%	Accepted
148	K → AG	46%	Rejected	695	KV → AGN	43%	Rejected
·	·	·	·	696	N → AGKV	50%	Rejected
461	FL → GS	60%	Rejected	697	NV → AGK	75%	Rejected
462	FLS → G	100%	Accepted	698	V → AGKN	16%	Rejected

***(2) Disease-2: Breast cancer*** In case of *Breast Cancer*, the algorithm handled the protein sequence of *Breast Cancer Type 1 Susceptibility* protein and generated total 1806 frequent itemsets of amino acids considering minimum support count 5. Here, total 20,884 association rules were generated from 1806 frequent itemsets. Among these, only 80 rules satisfied the minimum confidence level (90%) and were considered as accepted strong association rules and rest rules were rejected. Few of these accepted rules are shown in Table 4.

**Table 4:** Accepted strong association rules for *breast cancer* (not full list)

Ser	Assoc rule	Conf	Ser	Assoc rule	Conf
1	AD → E	100%	56	GKLN → P	100%
2	DH → E	90%	·	·	·
3	MS → E	93%	·	·	·
·	·	·	62	GQRS → L	100%
·	·	·	63	NQRS → L	100%
25	DRS → E	91%	64	LRSV → E	100%
26	DSV → E	100%	65	EKQV → L	100%
27	DNV → E	100%	·	·	·
28	FLN → P	100%	·	·	·
·	·	·	·	·	·
·	·	·	78	LNQST → P	100%
40	IKR → S	100%	79	GLQV → S	100%
41	FKV → S	90%	80	KQSV → L	100%

***(3) Disease-3: Cystic fibrosis*** Here, the algorithm handled the protein sequence of *Cystic Fibrosis Transmembrane Conductance Regulator (CFTR)* protein and generated total 1464 frequent itemsets of amino acids considering minimum support count 5. Total 14,792 association rules were generated from 1464 frequent item sets. Among these, only 96 rules satisfied the minimum confidence level (90%). Hence, these rules were considered as accepted strong association rules and rest rules were rejected. Few of these accepted rules are shown in Table 5.

**Table 5:** Accepted strong association rules for *cystic fibrosis* (not full list)

Ser	Assoc rule	Conf	Ser	Assoc rule	Conf
1	AG → L	90%	70	EKPQ → L	100%
2	DT → L	92%	71	LPQR → K	100%
3	HV → L	91%	72	ALQR → S	100%
4	NW → L	100%	·	·	·
5	TW → L	90%	·	·	·
6	AM → L	93%	82	HIKV → S	100%
7	PY → L	100%	83	HISV → K	100%
8	QY → L	92%	84	AGIS → L	100%
·	·	·	·	·	·
·	·	·	·	·	·
24	DTV → L	100%	94	APSV → L	100%
25	HTV → L	100%	95	LPST → V	100%
26	AIM → L	100%	96	IKLTV → S	100%

***(4) Disease-4: Nephrogenic diabetes insipidus*** Here, total 1152 association rules were generated from 234 frequent itemsets. Among these, only 54 rules satisfied the minimum confidence level (90%) and were considered as accepted strong association rules and rest rules were rejected. Few of the accepted rules are shown in Table 6.

**Table 6:** Accepted strong association rules for *nephrogenic diabetes insipidus* (not full list)

Ser	Assoc rule	Conf	Ser	Assoc rule	Conf
1	K → A	100%	32	AFG → P	100%
2	N → S	100%	33	FG → AP	100%
3	FW → A	100%	·	·	·
·	·	·	·	·	·
16	CV → A	100%	40	FPV → A	100%
17	FV → A	100%	41	GPV → A	100%
·	·	·	·	·	·
28	DE → P	100%	52	DLP → E	100%
29	FG → P	100%	53	AMT → L	100%
30	GI → V	100%	54	FLPV → A	100%

***(5) Disease-5: Retinitis pigmentosa 4*** Here, total 1252 association rules were generated from 268 frequent itemsets where only 49 satisfied minimum confidence level (90%) and were considered as accepted strong association rules and rest rules are rejected. A few of the accepted rules are shown in Table 7.

**Table 7:** Accepted strong association rules for *retinitis pigmentosa 4* (not full list)

Ser	Assoc rule	Conf	Ser	Assoc rule	Conf
1	W → A	100%	26	GIT → F	100%
2	W → L	100%	27	FTV → G	100%
·	·	·	31	GPT → F	100%
12	GM → F	100%	·	·	·
13	NY → P	100%	46	ALY → I	100%
·	·	·	47	AVY → I	100%
22	CY → V	100%	48	FNPT → G	100%
23	AFT → G	100%	49	GNPT → F	100%

### Useful association rules identification

The strong association rules obtained by the previous process were required to be evaluated by some measuring tools to identify useful strong association rules. Objective measuring tools *Lift* and *Improve* were used for this purpose [18]. However, *Lift* and *Improve* have some limitation as discussed in para II(*F*). Thus considering the effectiveness and stability in results, in this work (as mentioned earlier) improved objective measuring tools (i. e. *Bi-lift, Bi-improve* and *Bi-confidence*) were used to evaluate the association rules comprehensively.

*Lift, Bi-lift, Bi-improve* and *Bi-confidence* value of each of the association rules were calculated and finally only useful rules were sorted out based on the following criteria:

- The rule (*A* → *B*) will be considered as positively correlated rule (emergence of “*A*” promotes the emergence of “*B*,”) if its *Lift* value is greater than 1. Thus, those rules are useful only whose *Lift* value is greater than 1. The higher the *lift*(*A* → *B*) value, the better the rule (*A* → *B*) is, while the higher the (*Ā* → *B*) is, the worse the rule (*A* → *B*) is.
- The higher the *Bi-lift*(*A* → *B*) value, the better the rule (*A* → *B)* is.
- The higher the *Bi*-*improve*(*A* → *B*) value, the better the rule (*A* → *B)* is.
- If the *Bi-confidence* value is greater than 0, then *P(AB)* > *P(A)P(B)*, which shows that “*A*” and “*B*” have the positive correlation. Thus, those rules are useful only whose *Bi-confidence* value is greater than 0. The higher the *Bi*-*confidence* (*A* → *B*) value, the better the rule *A* → *B* is.

***Disease-1: Sickle cell anemia*** In case of *Sickle Cell Anemia*, 95 rules were considered as accepted strong association rules (as per previous step) which were further evaluated to determine their usefulness. In doing so, *Lift, Bi-lift, Bi-improve* and *Bi-confidence* values of each of these association rules were calculated and shorted out based on the criteria stated in the earlier paragraph. Finally 59 rules were selected as useful strong association rules (Table 6) and rest 36 rules were redundant or might be misleading and thus not effective (Table 8).

**Table 8:** Usefulness measures of association rules for *sickle cell anemia*

Ser	Rules	*Lift*	***Bi-lift***	***Bi-Improve***	***Bi-confidence***
*Useful strong association rules*					
1	GT → AN	3.75	12	0.183	0.917
2	GT → KN	3.75	12	0.183	0.917
3	AGT → KN	3.75	12	0.183	0.917
4	GKT → AN	3.75	12	0.183	0.917
5	GT → AKN	3.75	12	0.183	0.917
6	AN → GK	3	11	0.242	0.909
7	GS → FL	3	6	0.167	0.833
·	·	·	·	·	·
·	·	·	·	·	·
41	AGNV → K	1.364	1.5	0.067	0.333
42	FL → G	1.154	1.25	0.067	0.2
43	AN → G	1.154	1.222	0.048	0.182
44	KN → G	1.154	1.222	0.048	0.182
·	·	·	·	·	·
·	·	·	·	·	·
58	AKNT → G	1.154	1.2	0.033	0.167
59	AKNV → G	1.154	1.2	0.033	0.167
*Redundant rules*					
60	GH → A	1	1	0	0
61	GK → A	1	1	0	0
62	KN → A	1	1	0	0
·	·	·	·	·	·
·	·	·	·	·	·
94	PT → V	0.833	0.786	− 0.073	− 0.273
95	FG → L	0.833	0.769	− 0.1	− 0.3

In this case, the first accepted useful association rule is GT → AN as it satisfies the required criteria as shown below:

- **Criteria-1**: *Lift* value should be greater than 1.
- Test: Here, *lift* (GT → AN) = 3.75, which is greater than 1. So, criteria-1 is satisfied.
- **Criteria-2**: The higher the *Bi-lift*(*A* → *B*) value, the better the rule (*A* → *B)* is.
- Test: Here, *Bi-lift*(GT → AN) = 12, which is a positive higher value. So, criteria-2 is satisfied.
- **Criteria-3**: The higher the *Bi*-*impro*v(*A* → *B*) value, the better the rule (*A* → *B)* is.
- Test: Here, *Bi-improve*(GT → AN) = 0.183,which is a positive value. So, criteria-3 is satisfied.
- **Criteria-4**: *Bi-confidence* value is greater than 0.
- Test: Here, *Bi-confidence*(GT → AN) = 0.917, which is greater than 0. So, criteria-4 is satisfied.

***(2) Disease-2: Breast cancer*** Similarly, in case of *Breast Cancer*, *Lift, Bi-lift, Bi-improve* and *Bi-confidence* values of 80 accepted rules were calculated and evaluated. Finally 19 rules were selected as useful strong association rules and rest 61 rules were redundant or might be misleading and thus not effective (Table 9).

**Table 9:** Usefulness measures of association rules for *breast cancer*

Ser	Rules	***Lift***	***Bi-lift***	***Bi-Improve***	***Bi-confidence***
*Useful strong association rules*					
1	ANPT → G	2.149	2.235	0.018	0.552
2	NQST → P	1.948	2.011	0.016	0.503
3	FLN → P	1.948	2.0	0.013	0.5
4	GKLN → P	1.948	2.0	0.013	0.5
5	GLNT → P	1.948	2.0	0.013	0.5
6	LNQST → P	1.948	2.0	0.013	0.5
7	ILQS → N	1.545	1.569	0.01	0.363
8	IPSV → K	1.365	1.379	0.007	0.275
9	EKQV → L	1.199	1.208	0.006	0.172
10	DHP → L	1.199	1.207	0.005	0.171
11	QRT → L	1.199	1.207	0.005	0.171
12	GPST → L	1.199	1.207	0.005	0.171
13	GQRS → L	1.199	1.207	0.005	0.171
14	NQRS → L	1.199	1.207	0.005	0.171
15	DPY → L	1.199	1.205	0.005	0.17
16	DEHP → L	1.199	1.205	0.005	0.17
17	FPST → L	1.199	1.205	0.005	0.17
18	EKQSV → L	1.199	1.205	0.005	0.17
19	NQR → L	1.079	1.084	0.004	0.069
*Redundant rules*					
20	ADR → E	0.944	0.943	− 0.002	− 0.06
·	·	·	·	·	·
·	·	·	·	·	·
78	EGKV → S	0.835	0.829	− 0.008	− 0.206
79	EQR → S	0.751	0.741	− 0.017	− 0.315
80	FKV → S	0.751	0.741	− 0.017	− 0.315

***(3) Disease-3: Cystic fibrosis*** In case of *Cystic Fibrosis*, the algorithm handled the corresponding protein sequence and generated 96 accepted strong association rules. Basing on *Lift, Bi-lift, Bi-improve* and *Bi-confidence* values of these rules, finally 35 rules were sorted out as useful strong association rules and rest 61 rules were redundant or might be misleading and thus not effective (Table 10).

**Table 10:** Usefulness measures of association rules for *cystic fibrosis*

Ser	Rules	***Lift***	***Bi-lift***	***Bi-Improve***	***Bi-confidence***
*Useful strong association rules*					
1	EKLP → Q	2.209	2.328	0.023	0.57
2	PVW → A	1.783	1.833	0.015	0.455
3	CLR → A	1.783	1.833	0.015	0.455
4	HILV → T	1.783	1.833	0.015	0.455
5	HILS → T	1.783	1.833	0.015	0.455
6	FPR → V	1.644	1.707	0.022	0.414
7	FIPR → V	1.644	1.69	0.017	0.408
·	·	·	·	·	·
·	·	·	·	·	·
·	·	·	·	·	·
32	HKLV → S	1.203	1.212	0.006	0.175
33	IKLTV → S	1.203	1.212	0.006	0.175
34	IKLV → S	1.094	1.102	0.006	0.084
35	DIR → S	1.083	1.089	0.005	0.074
*Redundant rules*					
36	ANW → L	0.809	0.803	− 0.008	− 0.245
37	DET → L	0.809	0.803	− 0.008	− 0.245
·	·	·	·	·	·
·	·	·	·	·	·
94	EQR → L	0.728	0.714	− 0.024	− 0.361
95	APS → L	0.728	0.714	− 0.024	− 0.361
96	AG → L	0.728	0.698	− 0.053	− 0.389

***(4) Disease-4: Nephrogenic diabetes insipidus*** Similarly, *Lift, Bi-lift, Bi-improve* and *Bi-confidence* values of 54 accepted rules were calculated and evaluated. Finally 14 rules were selected as useful strong association rules (Table 11).

**Table 11:** Usefulness measures of association rules for *nephrogenic diabetes insipidus*

Ser	Rules	***Lift***	***Bi-lift***	***Bi-Improve***	***Bi-confidence***
*Useful strong association rules*					
1	DLP → E	3.455	4.857	0.084	0.794
2	FG → AP	2.375	2.833	0.068	0.647
3	GI → AV	2.235	2.615	0.065	0.618
4	CV → AL	1.9	2.125	0.056	0.529
5	AE → P	1.462	1.6	0.059	0.375
·	·	·	·	·	·
·	·	·	·	·	·
11	GI → V	1.267	1.308	0.025	0.235
12	AGI → V	1.267	1.308	0.025	0.235
13	N → S	1.086	1.103	0.015	0.094
14	AN → S	1.086	1.097	0.009	0.088
*Redundant rules*					
15	K → A	0.809	0.791	− 0.028	− 0.265
16	FG → A	0.809	0.791	− 0.028	− 0.265
·	·	·	·	·	·
·	·	·	·	·	·
53	PQ → L	0.776	0.75	− 0.044	− 0.333
54	MT → L	0.776	0.75	− 0.044	− 0.333

***(5) Disease-5: Retinitis pigmentosa 4*** In case of *Retinitis Pigmentosa 4*, the algorithm handled the protein sequence of *Rhodopsin (Opsin-2)* protein and generated 49 strong association rules. Here, basing on *Lift, Bi-lift, Bi-improve* and *Bi-confidence* values, all 49 rules were selected as useful strong association rules (Table 12).

**Table 12:** Usefulness measures of association rules for *retinitis pigmentosa 4*

Ser	Rules	***Lift***	***Bi-lift***	***Bi-Improve***	***Bi-confidence***
*Useful strong association rules*					
1	ALS → W	7	31	0.111	0.968
2	W → AL	3.5	6	0.119	0.833
3	PW → AL	3.5	5.167	0.092	0.806
4	SW → AL	3.5	5.167	0.092	0.806
5	QS → E	2.188	2.727	0.09	0.633
6	AFP → S	2.059	2.385	0.066	0.581
·	·	·	·	·	·
21	AVY → I	1.458	1.55	0.041	0.355
22	W → L	1.207	1.25	0.029	0.2
23	AW → L	1.207	1.25	0.029	0.2
24	CI → L	1.207	1.24	0.022	0.194
·	·	·	·	·	·
34	GPT → F	1.167	1.2	0.024	0.167
35	EM → F	1.167	1.192	0.018	0.161
36	MS → F	1.167	1.192	0.018	0.161
·	·	·	·	·	·
48	LSW → A	1.094	1.107	0.011	0.097
49	ILV → A	1.094	1.107	0.011	0.097

## Summary of the result

Considering the limitation of earlier studies, this work designed a uniform method to predict the patterns and association rules of the most dominating amino acids for different protein misfolded diseases. The support thresholds were kept relatively low to examine large amount of frequent patterns and their association rules. And the rules were then tested using improved objective measuring tools (*Bi-lift, Bi-improve and Bi-confidence*) to evaluate the association rules comprehensively. Finally following patterns and useful strong association rules of the most dominating amino acids for experimented protein misfolded diseases were found as outcome:

**Table:** 

***Disease-1: Sickle cell anemia***				
GT → AN	GT → KN	AGT → KN	GKT → AN	GT → AKN
AN → GK	GS → FL	NT → GK	KP → TV	ANT → GK
NT → AGK	ANV → GK	GT → N	AGT → N	GKT → N
AGKT → N	KP → T	GH → AL	GT → AK	NT → AK
KPV → T	GNT → AK	KN → AG	GS → F	FS → GL
GLS → F	NT → AG	KNT → AG	KNV → AG	AN → K
AT → K	AGN → K	GT → K	NT → K	AGT → K
ANT → K	GNT → K	ANV → K	ATV → K	AGNT → K
AGNV → K	FL → G	AN → G	KN → G	NV → G
AKN → G	ALV → G	AD → G	LN → G	FS → G
NT → G	AFL → G	FLS → G	ANT → G	KNT → G
ANV → G	KNV → G	AKNT → G	AKNV → G

**Table:** 

***Disease-2: Breast cancer***				
ANPT → G	NQST → P	QRT → L	GKLN → P	LNQST → P
IPSV → K	EKQV → L	NQR → L	GPST → L	EKQSV → L
DPY → L	DEHP → L	FPST → L	ILQS → N	NQRS → L
FLN → P	DHP → L	GLNT → P	GQRS → L

**Table:** 

***Disease-3: Cystic fibrosis***				
EKLP → Q	HISV → K	HIKV → S	HKLS → V	HKR → S
APW → V	ALQR → S	HILV → T	DKSV → I	IKLV → S
LPQR → K	HIKT → S	FILP → V	DIM → S	FIPR → V
ADKS → I	CLR → A	AFLV → I	IKLTV → S	FIN → K
AIKN → S	PRT → V	DIKV → S	FPR → V	FGQ → I
PVW → A	DLRS → I	HKLV → S	LPST → V	ADN → S
AQW → V	HKV → S	HILS → T	FMR → I	DIR → S

**Table:** 

***Disease-4: Nephrogenic diabetes insipidus***						
DLP → E	GI → AV	FG → P	AE → P	DE → P	GI → V	DEL → P
AFG → P	AGI → V	AE → P	CV → AL	N → S	AN → S	FG → AP

**Table:** 

***Disease-5: Retinitis pigmentosa 4***					
ALS → W	W → AL	NY → P	GT → F	LSW → A	APW → L
FTV → G	EM → F	QV → T	AFT → G	AFS → P	ASW → L
ALY → I	FH → T	AW → L	FGI → T	FGY → T	GNPT → F
PW → L	SW → L	GM → F	FNP → G	GNT → F	GMN → F
GTY → F	LW → A	QS → E	ILV → A	SW → AL	
GPT → F	W → A	AY → I	AGT → F	PW → AL	
FNP → G	KV → T	W → L	SW → A	LPW → A	
AVY → I	MS → F	H → T	GIT → F	GTV → F	
AFP → S	PW → A	CY → V	CI → L	MNT → F	

**Fig. 9:**
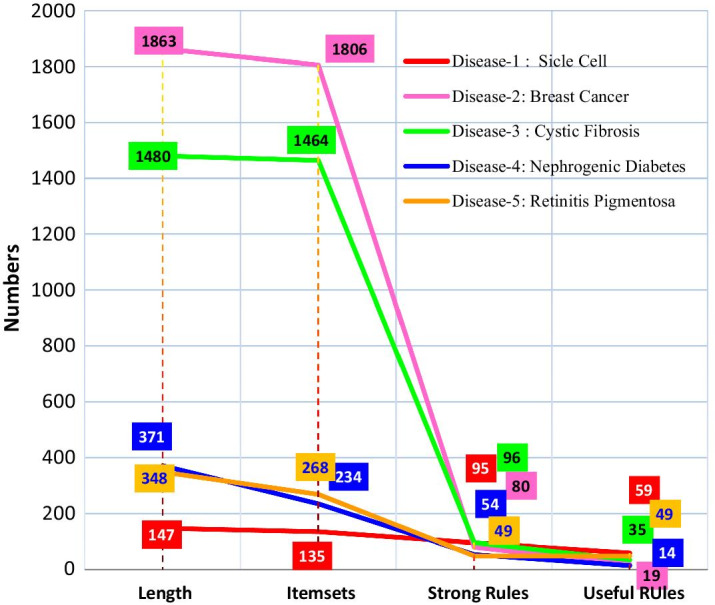
Summary of the lengths, itemsets and the rules for the protein sequences of associated diseases

This work initially generated 135, 1806, 1464, 234 and 268 itemsets from the corresponding protein sequences of *Sickle Cell Anemia, Breast Cancer, Cystic Fibrosis, Nephrogenic Diabetes Insipidus (NDI),* and *Retinitis Pigmentosa 4 (RP4),* respectively. Then the algorithm generated association rules from those itemsets. The association rules which fall below the threshold Confidence (90%) were pruned as strong association rules. After using objective measuring tools over these strong association rules, the final useful rules were found to be only 59, 19, 35, 14 and 49. These final rules indicate the most dominating amino acids and their patterns for *Sickle Cell Anemia, Breast Cancer, Cystic Fibrosis, Nephrogenic Diabetes Insipidus (NDI),* and *Retinitis Pigmentosa 4 (RP4*) disease (Fig. 9).

## Comparison with previous studies

It has been already mentioned that all the previous studies, in this aspect, were focused to predict the pattern and association rules of the most dominating amino acids which were associated with *Chromaffin Tumor* disease only. As per the literature [2, 5, 6], following are the accepted strong association rules as generated for *Chromaffin Tumor* disease:

- *PN → L* [2]
- *PI → K* [2, 6]
- *I → K* [5]
- *V → L* [5]

In this work, the same protein sequence (involved with *Chromaffin Tumor* disease) was tested and the result is shown in Table 13.

**Table 13:** Useful strong association rules for *chromaffin tumor* disease (min support count = 5)

Ser	Rules	***Confidence***	***Lift***	***Bi-lift***	***Bi-improve***	***Bi-confidence***
1	F → D	100%	1.75	2.091	0.093	0.522
2	DN → L	100%	1.12	1.15	0.023	0.130
3	PN → L	100%	1.12	1.15	0.023	0.130
4	PI → K	100%	1.12	1.15	0.023	0.130
5	KLY → P	100%	2.00	2.556	0.109	0.609

From this table is evident that *PN → L* and *PI → K* rules as generated by the literature [2, 5, 6] are useful strong association rules and *I → K* and *V → L* are redundant and should be thus rejected. On the other hand *F → D, DN → L* and *KLY → P* are useful strong association rules which were discarded by the literature.

## Implication of the findings

Patterns in protein sequences possess multifarious importance. Pattern identification can be used for predicting protein functions, protein fold (structure) recognitions, protein family detection, multiple sequence alignment, etc. Moreover, protein patterns can be used to predict the functions of newly discovered or unknown proteins or to screen genomic databases for other proteins with similar functionality [23]. This work is focused to predict the pattern and association rules of the most dominating amino acids in the protein sequences associated with particular protein misfolded diseases.

Thus identification/reporting of such variant of amino acids for those particular five genetic diseases may have versatile implications. Some implication of such findings are related to medical science, some are concerned to Genetics, Bioinformatics and Biotechnology or some are of Protein Sequencing Research as highlighted below:

- It can be applied for gene study through DNA sequencing, thus particular mutation can be edited through research.
- With the information of such data mining, prenatal diseases can be identified,
- An improved capacity in identifying the relations among the most dominating amino acids in protein sequences related to disease will have an immediate impact on the diagnosis, treatment, and prevention of genetic disorders. As more population-based data are accumulated, amino acids based diagnosis will become more common and the potential for somatic cell gene therapy will increase. Furthermore, the availability of molecular probes for specific gene loci will permit detection of the carriers of disease-associated genes. (G. N. N. Sultana, personal communication, Jun 23, 2019)
- Overall, in addition to the treatment action, such data gives the physicians to take the necessary genetic counselling. Thereby this work may open up new opportunities in medical science to handle genetic disorder diseases.
- Disease susceptibility can be predicted through most dominating amino acid changes.
- Understanding the complex interplay between genes and proteins requires integration of data from a wide variety of sources, i.e. gene expression, genetic linkage, protein interaction, and protein structure among others. Thus, this database can become critical for the integration, representation and visualization of heterogeneous biomedical data. (G. N. N. Sultana, personal communication, Jun 23, 2019)
- Biotechnologically, such data might allow development of new drugs for treatment and tools/biomarker for disease diagnosis.
- Identifying the relations among the most dominating amino acids in protein sequences can be implemented by focusing on how a protein leads to the heritable form of the respective disease. So research on understanding the normal function of genetically associated proteins in such diseases can be marginalized the complex roles of these proteins play in their respective disorders.
- In our work, we partitioned the whole amino acids sequence into sub sequences of length ten to find association rules. This type of consideration has the shortcoming of losing the support count of association rules in the border of window. However, for making the computational tasks easier we have considered the partitioning of length ten. This type of partitioning problem can be solved using windows overlapping. Another approach can be the used of random partition windows. In this case, for each rule, the bias in the border of window will be averaged via the average support count of many times of partition, so that the bias can be ignored approximately. Due to computational costs in this paper, we do not consider these two solutions. In fact, there is a trade-off between the fixed length partitioning and other two ways of partitioning. In future, we plan to test the performance considering two other above mentioned scenarios.

## Conclusion and future work

### Conclusion

Protein, being an integral part of every living organism, if not folded properly may cause critical genetic diseases. As amino acids are the building blocks of protein, relationship among the dominating amino acids and identification of their patterns is an important issue. This work focused to recognize frequent patterns among five complex protein misfolded genetic disorder human diseases and the relationship of the dominating amino acids using association rule mining. In doing so, itemsets and association rules were generated from the protein sequences. These rules were further evaluated and sorted out with objective measuring tools so that the only strong and interesting patterns are obtained. However, the proposed algorithm may be used to identify pattern of amino acids from associated proteins of other diseases also.

Patterns in protein sequences usually have functional, structural or family classification importance. Pattern identification can be used for predicting protein functions, protein fold (structure) recognitions, protein family detection, multiple sequence alignment, etc. The patterns acquired from this work are quite impressive. In addition to the above usual applications, an improved capacity in identifying the relations among the most dominating amino acids in protein sequences related to disease will have an immediate impact on the diagnosis, treatment, and prevention of protein misfolded diseases. And thereby this work may open up new opportunities in medical science to handle genetic disorder diseases.

### Future work

In this work, only five protein misfolded diseases were experimented. Again, protein sequence length of some of the diseases was relatively small. However, in future, more complex protein misfolded diseases and associated with larger length of protein sequences may be considered for experimentation. On the other hand, in this work Apriori algorithm was used as a pattern mining technique for association rule mining. However, as a newer method, Fuzzy Association rule mining technique may be adopted to generate more reliable association rules and test accordingly. In this work, the protein sequences were partitioned into subsequences of length 10. If the length of the subsequences is changed, the generated rules may also be changed. As such, rules can be generated considering the length as 10, 15, 20,.... and thereafter only the common rules between each list can be sorted out. Generating rules in this way may have better potentiality and validity.

## Data Availability

Not applicable.
